# Autonomic changes induced by pre-competitive stress in cyclists in relation to physical fitness and anxiety

**DOI:** 10.1371/journal.pone.0209834

**Published:** 2018-12-27

**Authors:** Iransé Oliveira-Silva, Vinícius Araújo Silva, Raphael Martins Cunha, Carl Foster

**Affiliations:** 1 Department of Physical Education, University Center of Anápolis-UniEVANGÉLICA, Anápolis, Brazil; 2 Department of Exercise and Sport Science, University of Wisconsin, La Crosse, Wisconsin, United States of America; University of Rome, ITALY

## Abstract

Cycling is a sport which requires great physical effort from athletes. The stress and anxiety of competition might interfere greatly with performance, by impacting the autonomic system. Physiological alterations, such as situations that irritate, frighten or excite the individual can cause a stress response, defined as a response of the organism to reestablish the homeostasis, regardless of its relationship to a sports situation. The aim of this study was to present data on the autonomic changes induced by precompetitive stress in cyclists and their relation with physical fitness and anxiety. Twelve healthy cyclists aged between 18 and 40 years, with previous experience in competition at the regional level, participated in the study. Heart rate variability (HRV) and physical fitness (body mass index, body fat and aerobic capacity) were measured 5 days before the Mountain Bike championship and remeasured 45 minutes before the start of the race (HRV and Anciety). Paired T test, effect size and correlational test were used. Cycling competition is a stressful situation capable of altering autonomic and hemodynamic parameters. We observed the increase in SDNN, reflecting an increase in sympathetic autonomic control. There were correlations between physical fitness with some autonomic parameters, while anxiety correlated with the years of experience in competition.

## Introduction

Cycling is a sport which requires great physical effort from athletes [1]. Competitive performance depends on physiological factors [2, 3], technical capabilities [4] and psychological factors [2]. In this sense, stress and anxiety might interfere greatly with performance, by impacting autonomic function [5]. Physiological alterations, such as situations that threaten, irritate, frighten or excite the individual can cause a stress response [6], defined as a response of the organism to reestablish homeostasis [7], regardless of whether it is related to ordinary life and / or sports situation.

Some studies have demonstrated the negative effects of stress on athletes in various sporting modalities [8–11]. Stress of this type also tends to negatively influence performance. There is also evidence that previous experience and physical fitness may modulate the stress response [2, 10–13].

One noninvasive way to evaluate autonomic control is through the analysis changes that occur between heart beats, known as heart rate variability (HRV). HRV is a technique that has been used in the clinical and sports environment, and is attractive because of its’ low cost and easy applicability [14, 15].

The analysis of HRV reflects the balance of autonomic control between the sympathetic and parasympathetic nervous systems [15], and is indicative of adaptive capacity, as HRV increases (i.e. desirable condition) [16] when stress is low. HRV tends to decrease with age [17], when there are pathogenic processes, in the presence of drug use [18], during pre-competitive stress [10], during overtraining [19], and before competitive matches [20].

Some evidence demonstrates that previous experience with stressful situations may minimize autonomic changes [12, 21]. This is why prior training and competition history is of such importance. Body composition and aerobic capacity also seem to influence autonomic control, where lower body fat and greater aerobic capacity are related to a better autonomic control [12, 22]. Cyclists, with very high aerobic capacities (i.e. ≥70ml kg min) [23] typically have very good autonomic control [24, 25].

In mountain biking, there are few studies that relate the stress caused by competition to the athlete's physical condition or to the effect of pre-competition anxiety on autonomic control. Therefore, the aim of this study was to present data on the autonomic changes induced by precompetitive stress in mountain cyclists and their relation with physical fitness and anxiety. It was hypothesized that cyclists during the precompetitive period would exhibit a lower HRV on the day of the competition compared to a control day, and that athletes with greater physical fitness would exhibit the smallest reduction in HRV during the day of the competition, and present the least evidence of anxiety.

## Materials and methods

Twelve healthy cyclists were recruited through of the Cycling Federation of Goiás. All athletes had previous experience in competition at the regional level, and participated in all stages of the evaluations. Table 1 shows the participants characteristics.

**Table 1:** Sample characteristics.

	[*X* (SD)]	Min-Max
Age (yrs)	27.5 (6.5)	18–40
Body mass (kg)	65.1 (7.6)	54.5–77.5
BMI (kg/m^2^)	21.1 (2.2)	19.4–28.1
Body Fat (%)	9.9 (4.6)	4–16
VO_2max_ (ml/kg/min)	64.2 (7.5)	51.2–74.5
HR rest. (bpm)	61 (10)	48–71
Cycling Experience (yrs)	7.12 (4.3)	0.5–15

Everyone involved in this study was informed about the procedures, and provided written informed consent. This research was approved by the Ethics Committee of UniEVANGÉLICA (protocol 1.968.437), in accordance with the principles of the Declaration of Helsinki.

The participants were between 18 and 40 years; did not present with any acute or chronic disease; had normal blood pressure (systolic BP ≤ 130 mmHg and diastolic BP ≤ 85 mmHg) pre-study; were systematically training; and were prepared to participate in the 2017 regional Mountain Bike cycling championship in Brazil.

### Study procedures

There were 2 evaluations for each athlete. The first was in the laboratory where the health of each participant was checked by a medical doctor (i.e. inclusion criteria), blood pressure was measured, following the recommendations of the Seventh report of the joint national committee on prevention, detection and treatment of high blood pressure [26], and heart rate variability (HRV) was measured under controlled circumstances. Physical fitness variables were also measured, including: body mass, height and body mass index (BMI), skinfolds [27] for define the percentage of body fat (BF), and aerobic capacity (VO_2max_) with Astrand 6-minute Cycle Test, according to Astrand et al. [28]. The second evaluation was in the competition room, before the regional championships, in the last race, of five stages of the regional circuit 2017. The race covered a total distance of 32 km, in Pirenópolis, Brazil. The level of difficulty was rated high, and the average time of the race was 82 minutes (66–98 min). All participants answered the questionnaire regarding anxiety (CSAI-2), and recorded heart rate variability, finalizing the records approximately 30 minutes before the competition, and before the start the warming up.

### Control and competition protocols

In the first lab visit, the control protocol (CP) had duration of ~ 30 minutes, and was performed at 8 am. It included measurement of blood pressure, HRV and physical fitness (body mass index, body fat and VO_2max_). The control protocol (CP) was completed 5 days before the Mountain Bike 2017 regional cycling championship, in the laboratory.

The competition protocol, had a duration of ~ 15 minutes, and was performed at 8 am, were all athletes had HRV measured in an identical manner to the control day. They also answered the questionnaire regarding anxiety (CSAI-2). These procedures were performed on the day of the regional Mountain Bike 2017 cycling championship, 45 minutes before the start of the competition, in a reserved place in the competition environment. Warm-up procedures were performed immediately following the HRV and CSAI-2 evaluations.

### Measures of HRV

The HRV measurements were performed using a heart rate analyzer (RS800, Polar Electro Oy, Finland), validated against electrocardiographic data (ICC ≥ 0.8). The procedures followed the recommendations of the Task Force [14].

The transmitter was placed on the participants' chest at the xiphoid process. Participants rested for 5 minutes, and then HRV was recorded for 10 minutes, while the participants were seated. Parameters of HRV analyzed were: Mean and standard deviation of heart rate (HR) in beats per minute (bpm); mean RR interval (RR), square root mean of successive differences between normal RR intervals (RMSSD), standard deviation of NN intervals (SDNN), low frequency spectral (LF) component; high frequency spectral component (HF); low frequency/high frequency (LF/HF) ratio; measure of short-term variability beat to beat RR of the Poincaré plot (SD1); entropy of the sample (SampEn); which is an example of a short-term fractal scaling (α1) [12, 14, 29].

### Anxiety assessment

To evaluate the athletes' anxiety, the Brazilian instrument version titled "Competitive State Anxiety Inventory-2" (CSAI-2), was used at a single moment (on the competition day). This instrument is known to be effective in assessing anxiety in sports [30]. It is a questionnaire composed of 27 questions, on a four-point Likert scale, assigning a concept of 1 (nothing) to 4 (very) with the ability to quantify three dimensions: cognitive anxiety (questions 1, 4, 7, 10, 13, 16, 19, 22 and 25), somatic anxiety (questions 2, 5, 8, 11, 14 [negative question], 17, 20, 23 and 26) and self-confidence (questions 3, 6, 9, 12, 15, 18, 21, 24 and 27). HRV measurement took place after the anxiety measurements.

### Data analysis

The Statistical Package for Social Sciences (SPSS v22) was used to analyze the data, based on mean and standard deviation (± SD), statistical significance at p <0.05. To analyze the data distribution, the Shapiro Wilk test with Lilliefors correction was used. If the variables did not present normal distribution, they were normalized through the natural logarithm (Ln). The differences in HRV between the 95% confidence intervals were presented, taking into consideration the competition day (i.e. 2nd stage) and the previous control day (i.e. 1^st^ stage) indicating the lower and higher value of the difference between moments [31]. The changes were tested by paired Student T test, and the magnitude between days was assessed using effect size In order to verify the correlation between BF, VO_2max_, HRV, practice time and anxiety, Pearson correlations (r) and the regression value (R^2^) were used to demonstrate the ability of one variable to influence measures of autonomic control.

## Results

The autonomic changes between control and competition day are presented in Table 2. On the competition day, there was the increase in SDNN with Δ = 65.4% (p = 0.026) and in HR, Δ = 18% (p = 0.011). Although there were mean changes in other autonomic variables, none achieved statistical significance.

**Table 2:** Heart rate variability measures prior to control and competition day.

Variable	Control	Competition Day	Δ(%)	p	ES Between days
SDNN (ms)	75.95 ± 15.41	125.65 ± 40.65	65.4	0.026	0.62
HR (bpm)	61 ± 10	72 ± 9	18.0	0.011	0.50
RMSSD (ms)	59.93 ± 18.70	44.85 ± 17.46	25.1	0.349	0.39
LnLF	7.84 ± 0.58	7.96 ± 0.92	1.5	0.854	0.07
LnHF	6.93 ± 1.26	6.58 ± 1.50	5.0	0.937	0.12
LnLF/HF	1.03 ± 0.80	1.26 ± 1.20	22.3	0.340	0.11
SD1	52.60 ± 19.38	51.43 ± 21.43	2.2	0.630	0.02
SampEn	1.22 ± 0.24	1.05 ± 0.43	13.9	0.295	0.23
α1	1.18 ± 0.21	1.30 ± 0.28	10.1	0.308	0.23

HR: Mean and deviation of heart rate; RR: mean RR interval; RMSSD: square root mean of successive differences between normal RR intervals; SDNN: standard deviation of NN intervals; LnLF: natural log of the low frequency spetral component; LnHF: natural log of the high frequency component; LnLF/HF:natural log of relation the low/high frequency spetral componente; SD1: measure of short-term variability beat to beat RR of the Poincaré plot; SampEn: entropy of the sample; α1: short-term fractal scaling; Δ(%): Magnitudes of difference between days are expressed as mean percentage change; ES: Effect size.

Regarding the participants' anxiety scores, self-confidence, the variable was presented the highest sum of anxiety domains evaluated, as can be seen in Table 3, below.

**Table 3:** Anxiety scores.

	[*X* (SD)]	Mín-Máx
Cognitive Anxiety	17 (5)	9–25
Somatic Anxiety	10 (2)	6–17
Self-confidence	24 (7)	8–36

The participants' body composition correlated with the ΔSampEn (P = 0.05). The aerobic capacity also correlated with ΔRMSSD (P = 0.05). Cycling experience is a variable that is related to lower somatic anxiety (P = 0.04) and higher self-confidence (P = 0.03), Table 4.

**Table 4:** Correlation between physical fitness, anxiety with ΔHRV (r and p value).

	AGE	Cycling Experience	ΔSDNN	ΔRMSSD	ΔLnLF	ΔLnHF	ΔLnLF/HF	ΔSD1	ΔSampEn	Δα1
**Body Fat**	0.570 (0.50)	-0.238 (0.45)	-0.017 (0.95)	0.302 (0.34)	-0.524 (0.08)	0.293 (0.35)	-0.019 (0.95)	0.200 (0.53)	**-0.346** **(0.05)**	-0.140 (0.66)
**VO**_**2**_**max**	0.059 (0.85)	0.473 (0.12)	0.327 (0.30)	**-0.504** **(0.05)**	0.073 (0.82)	-0.097 (0.76)	0.090 (0.78)	0.000 (0.99)	0.234 (0.46)	0.151 (0.64)
**Cognitive anxiety**	0.362 (0.24)	0.218 (0.49)	0.223 (0.48)	-0.153 (0.63)	-0.347 (0.27)	0.386 (0.21)	-0.310 (0.32)	0.541 (0.07)	-0.158 (0.62)	-0.461 (0.13)
**Somatic anxiety**	-0.442 (0.15)	**-0.579** **(0.04)**	-0.303 (0.33)	-0.316 (0.31)	0.121 (0.70)	-0.197 (0.53)	0.206 (0.52)	-0.015 (0.96)	0.044 (0.89)	0.267 (0.40)
**Self-confidence**	0.428 (0.16)	**0.601** **(0.03)**	0.143 (0.65)	0.203 (0.52)	0.119 (0.71)	-0.092 (0.77)	0.045 (0.89)	-0.218 (0.49)	0.079 (0.80)	0.020 (0.95)

VO_2_max: aerobic capacity assessment; ΔSDNN: difference of standard deviation of NN intervals; ΔRMSSD: difference of square root mean of successive differences between normal RR intervals; ΔLnLF: difference of natural log of the low frequency spetral componente; ΔLnHF: difference of natural log of the high frequency component; ΔLnLF/HF: difference of natural log of relation the low/high frequency spetral componente; ΔSD1: difference of measure of short-term variability beat to beat RR of the Poincaré plot; ΔSampEn: difference of entropy of the sample; Δα1: short-term fractal scaling.

## Discussion

A cycling competition is a stressful situation capable of altering autonomic and hemodynamic parameters. In this study, we observed the increase of 65% in SDNN (p = 0.02) immediately prior to competition, which may reflect the increase in sympathetic autonomic control. A factor observed in a previous study [32] that used the secretion of salivary alpha-amylase (sAA) in endurance running (half marathon) and also characterized the increase of the adrenergic mechanism, which inflates the sympathetic activity.

This may impact other physiological parameters, such as HR (18% increase [p = 0.01]). There were significant correlations between the physical fitness with some autonomic parameters, demonstrating that a better aerobic capacity led to a less parasympathetic withdrawal in relation to the control day, while anxiety correlated negatively with the years of experience in competition. Previous studies have shown that competitive anxiety is a mental state that includes cognitive, somatic and emotional components which may decrease athletic performance [33, 34, 35, 36].

HRV changes have been demonstrated in previous studies in response to different acute exercise sessions, observing autonomic stress characterized by a vagal reduction and the increase in sympathetic nervous activity [37, 38], similar to what reported by a previous study that utilized other important stress-sensitive biomarkers (i.e. cortisol and sAA)[32]. However, in relation to the pre-competitive stress in high-performance cycling athletes, there is little evidence.

In our study, we found evidence of a decrease in parasympathetic activity, suggesting that a considerable amount of non-linear behavior is by this branch of ANS, because non-linear analysis can be used as a powerful tool for the description of biosynthetic characteristics, since they are able to reveal small differences in the behavior of the systems, similar to what was reported in previous studies [39].

Stress and anxiety are expected in a competitive situation [33, 40, 41] and may have been minimized by differentiated physical condition (BF 9.86 ± 4.60% and VO_2max_ 64.18 ± 7.49 ml/kg/min) [12]. The result of this competition stage would have little influence on the overall annual classification, and it did not qualify for another tournament. In this situation, anxiety did not correlate with HRV (all parameters evaluation), though, it was clear that the cycling experience is a determinant that characterizes the athlete's anxiety.

The more experienced the athlete (i.e. years of training), the less anxiety they appear to experience [41]. This finding reinforces previous studies that affirmed that experience with competition was a factor acting to minimize anxiety [41, 42].

The aerobic capacity showed a negative correlation with the change in the RMSSD (i.e. ΔRMSSD) (p = 0.05), demonstrating that physical fitness should be considered when evaluating autonomic control [12].

This study had some limitations which should be considered. Firstly, the competition phase chosen for evaluation was not the most important of the season. Recent research has shown that pre-competitive stress is more evident in situations where the result really matters to the athlete [10].

Secondly, the inability to subdivide the sample into two groups (i.e. experienced and beginners), it was a potential factor that influenced the results [43], although the majority of the group were experienced. And also, the interval between HRV collection time on the race day and the start of the competition (i.e. ~ 30 minutes).

## Conclusion

A cycling competition is a stressful situation capable of altering autonomic parameters, normally requiring a considerable increase in sympathetic drive. Other markers of autonomic control evident in HRV data did not seem to respond strongly in the control vs pre-competition comparison. Body composition and aerobic capacity, indicators of physical fitness, presented a different relationship with HRV, while anxiety correlated with athletes' experience. Therefore, cyclists and their coaches need to work harder to control these indicators (i.e. body composition and aerobic capacity), as well as broadening the participation of beginner athletes in important events in order to broaden their experience.

New studies might seek to clarify how much previous experience contributes to the autonomic control of athletes in a more stressful situation.

## Supporting information

S1 File Datasets collected. (XLSX)
